# Expression of minichromosome maintenance protein 2 as a marker for proliferation and prognosis in diffuse large B-cell lymphoma: a tissue microarray and clinico-pathological analysis

**DOI:** 10.1186/1471-2407-5-162

**Published:** 2005-12-20

**Authors:** Ellen C Obermann, Philip Went, Annette Zimpfer, Alexandar Tzankov, Peter J Wild, Robert Stoehr, Stefano A Pileri, Stephan Dirnhofer

**Affiliations:** 1 Institute of Pathology, University of Regensburg, 93053 Regensburg, Germany; 2 Institute of Pathology, University Hospital Basel, 4031 Basel, Switzerland; 3 Institute of Pathology, University of Innsbruck, 6020 Innsbruck, Austria; 4 Department of Urology, University of Regensburg, 93053 Regensburg, Germany; 5 Chair of Pathology and Unit of Haematopathology, University of Bologna, Italy; 6 Institute of Pathology, University Medical Center, Hamburg-Eppendorf, 20246 Hamburg, Germany

## Abstract

**Background:**

Minichromosome maintenance (MCM) proteins are essential for the initiation of DNA replication and have been found to be relevant markers for prognosis in a variety of tumours. The aim of this study was to assess the proliferative activity of diffuse large B-cell lymphoma (DLBCL) in tissue microarray (TMA) using one of the minichromosome maintenance proteins (Mcm2) and to explore its potential value to predict prognosis.

**Methods:**

Immunohistochemistry for Mcm2 was performed on TMAs constructed from 302 cases of DLBCL. A monoclonal mouse antibody was used after heat induced antigen retrieval. Mcm2 expression was scored quantitatively. Positivity for Mcm2 was defined as presence of nuclear expression of Mcm2 in greater than or equal to 40 % of tumour cells. A statistical analysis was carried out of the association of Mcm2 and the clinico-pathological characteristics.

**Results:**

Mcm2 expression was clearly evident in the nuclei of proliferating non-neoplastic cells and tumour cells. Positivity for Mcm2 was found in 46% (98/211) of analysable cases. A significant correlation existed between Mcm2 expression and presence of bulky disease (p = 0.003). Poor disease specific survival was observed in patients with DLBCL positive for Mcm2 expression in the univariate analysis (p = 0.0424).

**Conclusion:**

Mcm2 expression can be used to assess tumour proliferation and may be useful as an additional prognostic marker to refine the prediction of outcome in DLBCL.

## Background

In Western countries, diffuse large B-cell lymphoma (DLBCL) is the most common type of mature B-cell lymphomas with a frequency of approximately 30 to 40% [1]. DLBCL is an aggressive but potentially curable disease. However, only about 40 to 45% of patients are cured with combination chemotherapy [2]. Currently, the International Prognostic Index (IPI) is the generally accepted predictor of prognosis [3]. In order to avoid over-treatment of some patients and identify patients at high-risk for early relapse or poor response to standard treatment, individualised prediction of prognosis becomes more and more important.

A number of biological markers have been studied for their predictive potential, but none has become universally accepted [4-7]. Gene expression profiling has helped to identify at least two subgroups of DLBCL, one with a germinal centre signature and the other with an activated B-cell signature, which show distinct clinical outcomes [3,8].

The proliferative capacity of neoplastic cells is an important feature of growing tumours. Assessment of cell proliferation may provide both pathologists and clinicians with more objective prognostic information [9,10]. Expression of Ki-67 as assessed by immunohistochemistry has become the standard proliferation marker [11-13]. In lymphoid neoplasms, it is controversial whether Ki-67 is a reliable prognostic indicator [14-17]. In DLBCL, a high proliferation index has been associated with an unfavourable clinical outcome in some studies [18]. Despite the extensive use of Ki-67, its functional significance still remains unclear [19]. It has been suggested, that Ki-67 plays a role in the ribosome biosynthesis rather than being directly responsible for cell proliferation [20,21]. Therefore, detecting markers directly involved in DNA replication might be a more precise method to evaluate the proliferative behaviour of a tumour.

The minichromosome maintenance (MCM) protein family consists of six members of DNA-binding proteins [22]. MCM proteins stand at the end of many signalling pathways involved in cell proliferation [23]. They ensure that synthesis of DNA is initiated only once during each cell cycle. All six proteins are abundant throughout the cell cycle and are broken down rapidly on differentiation or more slowly in quiescence [24,25]. Expression is only observed in cycling cells, there is no expression in quiescent and differentiating cells [25-28].

Antibodies for detection of MCM proteins in routinely processed tissue specimen have been found superior to Ki-67 in defining the proliferative compartments in both normal and abnormal human tissues [27,29-32]. Immunohistochemical assessment of all six MCM proteins has been observed to produce similar results in a range of tissue sections [33]. MCM proteins have been promoted as markers for cancer screening, surveillance and prognosis [27,29-31,34-36]. We used a specific monoclonal antibody directed against Mcm2 and a previously validated tissue microarray (TMA) with tissue samples of a large number of DLBCL [37]. Tissue microarrays are highly efficient for the investigation of large series of neoplasms including lymphoma [37-39].

The aim of this study was to systematically investigate if the analysis of Mcm2 expression might provide a novel tool to assess the proliferation of DLBCL and predict clinical outcome in patients with this disease.

## Methods

### Construction of tissue microarrays and acquisition of clinico-pathologic data

Tissue microarrays were constructed as described previously [39,40]. The TMAs contained a total of 302 tissue samples from tumours, which had been classified prior to this study as diffuse large B-cell lymphomas according to the WHO classification [1]. All samples had been obtained at the time of diagnosis, before any treatment had been given. Four different TMAs were constructed, each containing tumour samples from different histopathologic institutions (Basel, Bologna, Innsbruck, Zurich). Clinical data of patients with DLBCL at time of primary diagnosis and follow-up had been obtained by reviewing the charts. Clinico-pathologic data of patients at time of diagnosis are detailed in table 1. Retrieval of tissue and clinical data was performed according to the regulations of the local institutional review board and data safety laws.

### Immunohistochemistry

Four-micrometre sections of the TMA blocks were cut to adhesive-coated slides (Instrumedics Inc, Hackensack, NJ, USA) and stained using standard procedures. Briefly, immunohistochemical studies utilized an avidin-biotin peroxidase method with diaminobenzidine chromatogen. After heat induced antigen retrieval (microwave oven for 30 min at 250 W) immunohistochemistry was carried out in a NEXES immunostainer (Ventana, Tucson, AZ). The following primary antibody was used: BM28 (mouse monoclonal, clone 46, BD Biosciences, San Jose, US, final dilution 1:3000). The dilution had been established using adequate controls. Negative controls were obtained by omitting the primary antibody. The slides were evaluated without knowledge of clinical data. At least 10% of cases were re-evaluated by a second observer. Only cases containing clearly recognisable tumour tissue were analysed. One-hundred cells were assessed in each tumour core and the percentage of positive cells (i.e. cells with a distinct staining) was calculated. If a biopsy core contained less than 100 cells as many neoplastic cells as possible were evaluated. If more than one core from the same tumour was available the results were averaged. Cases were defined positive for Mcm2 if ≥40 % of tumours cells showed distinct nuclear staining. The determination of cut-off levels was based on the analysis of the area under the receiver operating characteristic curve (AUROC) as described below and as detailed in table 2.

### Statistical analysis

Statistical analyses were performed using SPSS version 10.0 (SPSS, Chicago, IL). Differences were considered statistically significant if P values were <0.05. Receiver operating characteristic (ROC) curves by plotting sensitivity versus (1-specificity) were used to evaluate the diagnostic performance of parameters at various cut-off points. An AUROC closer to 1 indicates greater discriminatory power, whereas an AUROC of 0.5 denotes no diagnostic potential. The optimum cut-off was calculated as the maximum value of sensitivity multiplied by specificity. Sensitivity and specificity were calculated according to standard formulas for the cut-off that represented the best discrimination derived from the ROC curves. The cut-off value representing the best discrimination derived from the ROC analysis was chosen for categorization of metric variables (LDH (lactate dehydrogenase) and expression of Mcm2). A statistical association between clinico-pathologic parameters and Mcm2 expression was tested using a two-sided Fisher's exact test. Disease specific survival (DSS) curves were calculated using the Kaplan-Meier method with significance evaluated by two-sided log-rank statistics. For DSS analysis, patients were censored at the time of their last clinical follow-up appointment or at their date of death not related to the tumour. A stepwise multivariable Cox regression model was adjusted, testing the independent prognostic relevance of Mcm2 positivity. The limit for reverse selection procedures was P = 0.1. The proportionality assumption for all variables was assessed with log-negative-log survival distribution functions.

## Results

### Clinico-pathologic data

Clinical follow-up data were available for 123 of 302 (40.7 %) patients with a median follow-up period of 23.5 months (range 1 to 177 months). The median follow-up for censored patients was 28.5 months. Clinico-pathologic parameters associated with poor disease specific survival were Ann Arbor stage (p = 0.0026), IPI (p < 0.0001), and serum LDH ≥300 U/l (p = 0.0133) according to the univariate analysis. The optimum cut-off level for LDH had been established at 306 U/l (AUROC method) and a cut-off of 300 U/l was used for further statistical analysis. Age at diagnosis and gender of patients did not make an impact on DSS. Neither the presence of bone marrow involvement, extranodal involvement nor bulky disease at presentation was statistically relevant for DSS.

The results of the univariate analysis are presented in table 3.

### Immunohistochemistry

**Figure 1:**
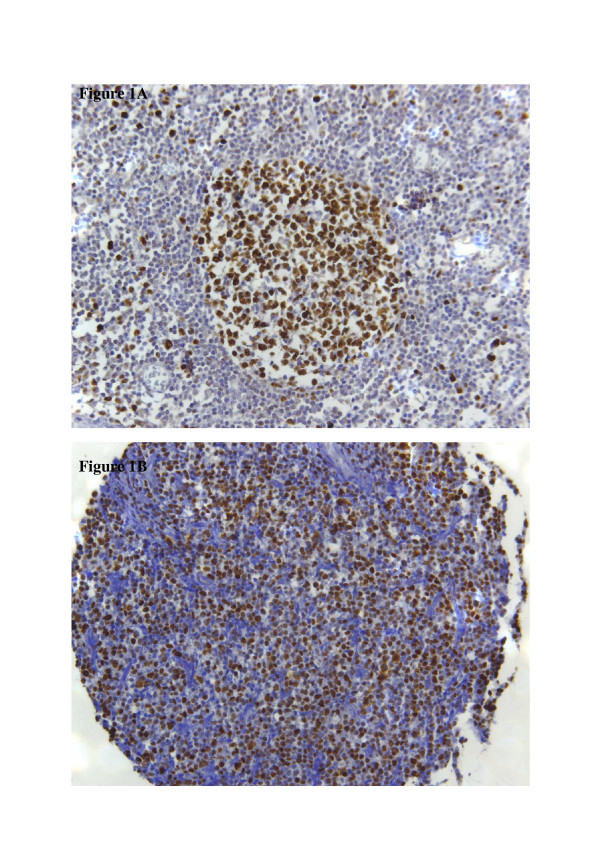
**Immunohistochemistry**. Figure 1A: Immunohistochemical staining of Mcm2 in a non-neoplastic lymph node. Mcm2-expression is found in the majority of cells in the germinal centre; only a few cells in the mantle zone are positive for Mcm2. Figure 1B: Immunohistochemical staining of Mcm2 in diffuse large B-cell lymphoma. This case is positive for Mcm2-expression with more than 40% of tumour cells expressing this marker.

Investigation of Mcm2 expression was informative in 69.9 % (211/302) of cases. Mcm2 expression in ≥40% of cells was detected in 46.4 % (98/211) of DLBCL. Mcm2 was clearly evident in the nuclei of tumour cells with low background staining. In non-neoplastic lymphoid tissue, which was used as a positive control and for establishing the appropriate staining protocols, expression of Mcm2 was mainly found in the germinal centres harbouring proliferating cells; expression in the mantle zone and parafollicular zone was negligible (Figure 1A). Identical results have previously been described [41].

The optimum cut-off for Mcm2 expression was calculated as 39.5 % of cells using the AUROC method. For practical reasons, a cut-off level of 40 % was chosen and patients were divided into two subgroups accordingly (positive: protein expression ≥40 % of cells; negative: protein expression <40 % of cells). For descriptive data analysis, all relevant variables were compared with Mcm2 expression (table 4). A representative image of immunohistochemical expression of Mcm2 in DLBCL is shown in figure 1B.

### Prognostic significance

**Figure 2:**
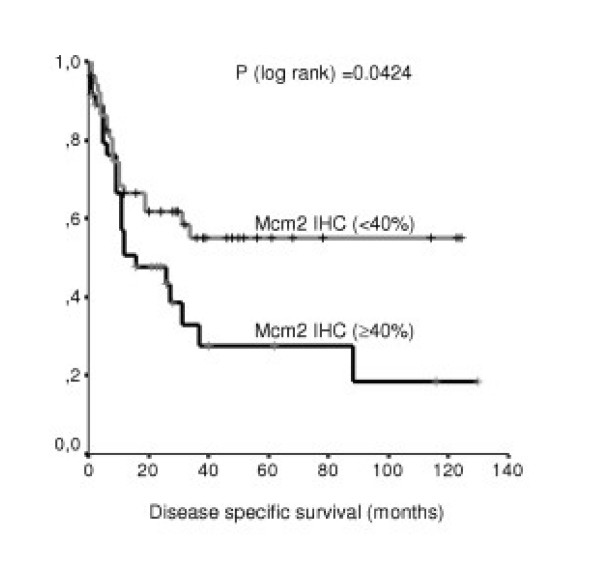
**Disease specific survival**. Distribution of time (months) to DLBCL-related deaths among patients with negative (<40 % of tumour cells) (n = 52) and positive (≥40% of tumour cells) (n = 36) expression of Mcm2 (Kaplan-Meier method). (IHC = immunohistochemistry)

Disease specific survival was compared between tumours positive and negative for Mcm2 by univariate log-rank statistics. Positivity for Mcm2 was associated with shorter DSS (p = 0.0424) (table 3; figure 2). However, when Mcm2 positivity was tested in the multivariate analysis in combination with LDH, IPI, and Ann Arbor stage it did not retain its prognostic value (p = 0.546). LDH was not found to be of prognostic relevance in the multivariate analysis as well (p = 0.063). Both IPI (p = 0.010) and Ann Arbor stage (p = 0.025) were significant prognostic markers in the multivariate analysis with respect to disease specific survival.

## Discussion

Cell proliferation is essential for the growth of any type of malignancy. Analysis of cell proliferation applying antibodies specifically detecting members of the MCM protein family has been proposed as a novel method for cell cycle assessment which may be of diagnostic and prognostic value in the histopathologic assessment of neoplasms. Other proliferation markers such as the widely used Ki-67 and proliferating cell nuclear antigen (PCNA) are probably not as effective as MCM proteins for this purpose, as Ki-67 is absent in early G1-phase, its function still remains unknown [42] and PCNA is less specific for determining proliferation since it is also present during DNA repair processes [43].

Analysis of MCM proteins by means of immunohistochemistry has been shown to be of prognostic value in a diverse range of human malignancies [30,34,44-51]; in lymphoma however, high expression of Mcm2 has been described in DLBCL, but so far data have never been evaluated with respect to clinical outcome [41].

Therefore we focused on the evaluation of Mcm2 expression in a large collective of clinically well documented patients with DLBCL using tissue microarray technology and immunohistochemistry. 46.4% of DLBCL showed positivity for Mcm2 defined as expression in ≥40% of tumour cells. Positivity was significantly associated with shorter disease specific survival in the univariate analysis (p = 0.0424). However, when tested in the multivariate analysis, it did not retain its prognostic value (p = 0.546). This finding may be attributable to the fact, that besides proliferation, a number of other factors not included into the clinico-pathological parameters of our analysis (such as concomitant morbidity) may influence disease specific survival. Therefore, assessment of Mcm2 expression may still be useful as an additional prognostic marker in conjunction with other established prognostic parameters.

## Conclusion

In summary, expression of Mcm2 in ≥40% of tumour cells was found to be a negative prognostic marker for disease specific survival in this large series of DLBCL. Protein expression of MCM proteins can easily be evaluated in routinely processed tissue specimen using specific antibodies in daily routine practice. Assessment of MCM protein expression may be used as a marker of proliferation as well as a potentially prognostic indicator, and further work in this area is warranted.

## Competing interests

The author(s) declare that they have no competing interests.

## Authors' contributions

ECO conceived and designed the study, was responsible for immunohistochemical studies and drafted the manuscript. PW, AZ, AT and SAP were responsible for acquisition of material and clinical data as well as construction of tissue microarrays. PJW performed the statistical analysis, helped to prepare figures and tables and interpreted the data. RS revised the article critically. SD participated in the design and coordination of the study. All authors read and approved the final manuscript.

## Pre-publication history

The pre-publication history for this paper can be accessed here:



## Figures and Tables

**Table 1:** Details of clinico-pathologic data and expression of Mcm2 in diffuse large B-cell lymphomas

**Variable**	**Categorisation**	**n analysable**	**%**
***Clinico-pathologic data:***			
Age at diagnosis			
	median 67 years (interquartile range 54–75 years)		
	<65 years	69	43.1
	≥65 years	91	56.9
Gender			
	male	97	53.3
	female	85	46.7
Ann Arbor stage			
	1	27	17.3
	2	50	32.1
	3	34	21.8
	4	45	28.8
International prognostic index (IPI)			
	0	2	1.3
	1	66	43.4
	2	29	19.1
	3	32	21.1
	4	23	15.1
Bone marrow involvement			
	yes	44	28.6
	no	110	71.4
Extranodal involvement			
	yes	54	35.8
	no	97	64.2
Bulky disease			
	yes	94	64.4
	no	52	35.6
Chemotherapy			
	no therapy	7	4.6
	CHOP	69	45.1
	other	77	50.3
Serum levels of LDH at diagnosis			
	median 306 U/l (interquartile range 185–443 U/l)		
	<300 U/l	61	49.2
	≥300 U/l	63	50.8
***Immunohistochemistry:***			
Mcm2 immunoreactivity			
	median 38% (interquartile range 29%–48%)		
	<40%	113	53.6
	≥40%	98	46.4

**Table 2:** Performance of metric variables in prediction of mortality using the AUROC (area under the receiver operating characteristic curve) method

	**Mcm2 IHC^c^**	**LDH^d^**
Optimum cutoff value	39.5%	306 U/l
Sensitivity^b^	0.512	0.620
Specificity^b^	0.696	0.476
AUROC^a^	0.582	0.553
95% confidence interval	0.462–0.702	0.434–0.672
p (asymtptotic significance)	0.182	0.384

^a ^AUROC, area under the receiver operating characteristic curve.

^b ^sensitivity and specificity were calculated for the cutoff, which represented the best discrimination as derived from the ROC curves.

^c ^IHC: immunohistochemistry

^d ^LDH: lactate dehydrogenase

**Table 3:** Univariate analysis of factors regarding disease specific survival

		**Tumor-related death**		
**Variable**	**Categorisation**	**n**	**events**	**p^a^**
***Clinico-pathologic data:***				
Age at diagnosis				
	<65 years	44	20	0.0805
	≥65 years	77	43	
Gender				
	male	64	31	0.4220
	female	59	34	
Ann Arbor Stage				
	1	19	6	**0.0026**
	2	37	16	
	3	27	15	
	4	39	27	
International prognostic index				
	0	2	0	**<0.0001**
	1	46	16	
	2	24	11	
	3	25	20	
	4	22	16	
Extranodal involvement				
	no	47	24	0.9997
	yes	70	36	
Bulky disease				
	no	73	36	0.3135
	yes	39	20	
Serum levels of LDH at diagnosis				
	<300 U/l	38	19	**0.0133**
	≥300 U/l	54	31	
***Immunohistochemistry:***				
Mcm2 immunoreactivity				
	<40%	52	21	**0.0424**
	≥40%	36	22	

^a ^Log rank test; bold face representing significant data.

**Table 4:** Clinico-pathologic and immunohistochemical parameters in relation to Mcm2 immunoreactivity

		**Mcm2 immunoreactivity**			
		
**Variable**	**Categorisation**	**n analysable**	**negative (<40%)**	**positive (≥40%)**	**p^a^**
***Clinico-pathologic data:***					
Age at diagnosis					
	<65 years	47	27	20	1.000
	≥65 years	63	37	26	
Gender					
	male	72	39	33	0.593
	female	57	34	23	
Ann Arbor Stage					
	1	15	10	5	0.342
	2	34	15	19	
	3	23	14	9	
	4	35	22	13	
International prognostic index					
	0	2	2	0	0.122
	1	48	27	21	
	2	21	14	7	
	3	20	13	7	
	4	14	4	10	
Bone marrow involvement					
	no	72	39	33	0.414
	yes	35	22	13	
Extranodal involvement					
	no	40	27	13	0.103
	yes	62	31	31	
Bulky disease					
	no	62	44	20	**0.003**
	yes	35	13	22	
Serum levels of LDH at diagnosis					
	<300 U/l	42	23	19	0.505
	≥300 U/l	41	26	15	

^a ^two-sided Fisher's exact test; bold type representing significant data.
